# Co-Cr-Fe-Mn-Ni Oxide as a Highly Efficient Thermoelectric High-Entropy Alloy

**DOI:** 10.1021/acsomega.2c08278

**Published:** 2023-04-14

**Authors:** Daria Pankratova, Silvia Maria Giacomelli, Khabib Yusupov, Farid Akhtar, Alberto Vomiero

**Affiliations:** † Department of Engineering Sciences and Mathematics, Luleå University of Technology, 97187 Luleå, Sweden; ‡ Department of Industrial Engineering, Università degli Studi di Padova, Via Giovanni Gradenigo, 6a, 35131 Padova PD, Italy; § Department of Physics, Chemistry, and Biology, Linköping University, 581 83 Linköping, Sweden; ∥ Department of Molecular Sciences and Nanosystems, Ca’ Foscari University of Venice, Via Torino 155, 30172 Venezia Mestre, Italy

## Abstract

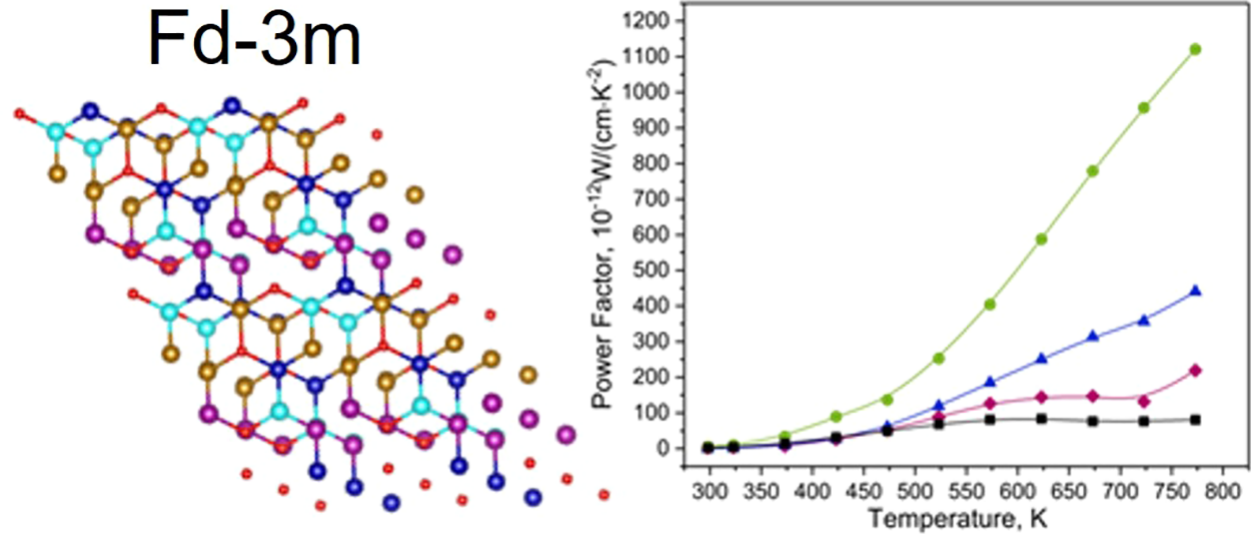

Among the existing materials for heat conversion, high-entropy alloys are of great interest due to the tunability of their functional properties. Here, we aim to produce single-phase high-entropy oxides composed of Co-Cr-Fe-Mn-Ni-O through spark plasma sintering (SPS), testing their thermoelectric (TE) properties. This material was successfully obtained before via a different technique, which requires a very long processing time. Hence, the main target of this work is to apply spark plasma sintering, a much faster and scalable process. The samples were sintered in the temperature range of 1200–1300 °C. Two main phases were formed: rock salt-structured *Fm*3̅*m* and spinel-structured *Fd*3̅*m*. Comparable transport properties were achieved via the new approach: the highest value of the Seebeck coefficient reached −112.6 μV/K at room temperature, compared to −150 μV/K reported before; electrical properties at high temperatures are close to the properties of the single-phase material (σ = 0.2148 S/cm, σ ≈ 0.2009 S/cm reported before). These results indicate that SPS can be successfully applied to produce highly efficient TE high-entropy alloys in a fast and scalable way. Further optimization is needed for the production of single-phase materials, which are expected to exhibit an even better TE functionality.

## Introduction

The energy demand is increasing constantly.^1^ Considering the environmental issues related to the use of fossil fuels, this demand needs to be fulfilled using renewable energy resources, like, for instance, solar, wind, and water energy. Among all, the reuse of wasted heat through thermoelectric (TE) materials is very appealing. Among the mentioned examples, TE technology is an attractive option because it can be easily used wherever a temperature difference is present.^2^ Other advantages of TE materials are their small operational size, which does not require large surface areas to be effective, and the lack of parts in motion or gas emissions.^3,4^

TE materials are used for the direct conversion of waste heat energy into electrical power. They have potential applications for waste heat recovery from different energy sources, such as power generation in deep space, solid-state cooling, portable and wearable electronics, etc.^5,6^

The efficiency of TE materials is described by the figure-of-merit *ZT*:
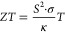
where *S* is the Seebeck coefficient, μV/K, σ is the electrical conductivity, S/cm, *T* is the temperature, K, and κ is the total thermal conductivity, W/(m·K).^7,8^ To achieve a high *ZT*, materials are required to have high values of Seebeck coefficient *S*, high electrical conductivity σ, and low thermal conductivity κ. The commercialization threshold for TE materials is *ZT* = 3.^4^

The most common TE materials are oxyselenides,^9−11^ Heusler alloys,^12^ etc. Among the materials, the highest TE yield is found for PbTe-based^13^ and Bi_2_Te_3_-based^14^ materials; however, Pb-based or any toxic-based materials should be avoided due to their environmental impact. An alternative to such materials could be found in multielemental systems or high-entropy alloys (HEAs).

Recent studies show that multicomponent oxide systems with high configuration entropy can exhibit impressive electrical, thermal, catalytic, or magnetic properties. As a result, high-entropy oxides (HEOs) are considered the alternative to conventional materials for different fields, such as microelectronics, catalytic converters, and energy and data storage.

HEAs are materials with a highly disordered structure that can help improve thermoelectric performance, by manipulating the electronic structure. This class of materials is defined as a system with 5 or more components, each having a concentration within the 5–35 at. % range. There are other advantages compared to conventional systems such as high entropy helping to stabilize solid solution-type structures.^15^ This concept has been used in different groups of materials, including HEOs,^16−20^ diborides (HEBs),^21^ and carbides (HECs).^22,23^

In the past few years, considerable research was performed on HEOs with different chemical compositions, such as (Gd_0.2_Nd_0.2_La_0.2_Sm_0.2_Y_0.2_)CoO_3_,^24^ Co-Cr-Fe-Mg-Mn-Ni-O,^20^ Sr(Ti_0.2_Fe_0.2_Mo_0.2_Nb_0.2_Cr_0.2_)O_3_,^18^ (Ca_0.2_Sr_0.2_Ba_0.2_Pb_0.2_La_0.2_)TiO_3_,^17,25^ Pb_0.99–*y*_Sb_0.012_Sn_*y*_Se_1–2*x*_Te_*x*_S_*x*_ (*x* = 0.25, *y* = 0.05, 0.10, 0.20, and 0.30 and *y* = 0, *x* = 0, 0.10, 0.20, and 0.25).^26^ The highest reached *ZT* for those materials is ∼2.^26^

One of the downsides of the materials described above is the required synthesis time. For most of the chemical compositions from the literature, the time for synthesis is 15–50 h. Hence, there is a need to simplify or shorten the obtaining process for such systems.

In the present work, we report on the synthesis of a HEO material with the chemical composition Co-Cr-Fe-Mn-Ni-O prepared via spark plasma sintering (SPS). Previously, Stygar et al.^20^ used a 20 h sintering process to prepare such a material, whereas in our approach, this was replaced with the SPS method, which significantly reduced the time from 20 h to less than 1 h.

The transport and structural properties were thoroughly studied, and the influence of the obtained parameters demonstrates that the SPS method is potentially beneficial for HEA synthesis. This study emphasizes that obtaining a two-phase HEO material with good TE properties in less than 1 h is possible. A further tweak of parameters should lead to the formation of a single-phase HEO, further improving the TE functionality.

## Experiments and Methods

### Synthesis

The alloys were prepared through the SPS method. The compositions of the alloys were determined by adjusting the relative fraction of the components in the precursor powders (Co_3_O_4_ (Sigma-Aldrich, <10 μm), Cr_2_O_3_ (Sigma-Aldrich, ≥98%), Fe_2_O_3_ (KeBo, no more information was given), MnO (Sigma-Aldrich, 60 mesh, 99%), and NiO (Sigma-Aldrich, 325 mesh, 99%)). The powders were manually ground for 10 min. After that, the powders were placed in a 14 mm-diameter graphite die and sintered via SPS (SPS-211Lx SPS Dr. SINTER LAB Jr. SERIES, Fuji Electronic Industrial) under a uniaxial pressure of 38 MPa under vacuum (1.6 × 10^1^ Pa). A series of samples were synthesized by varying the temperature from 1200 to 1300 °C during SPS, labeled according to Table 1. The highest temperature during the process was chosen below the melting temperature of almost all reagents. One of the precursors, Co_3_O_4_, has a melting point of 895 °C. This oxide is the mix of two oxides CoO and Co_2_O_3_. An important note is that the melting temperature (Table S1) for the precursors can be lower in vacuum and under pressure. To prevent the leakage of the sample around the melting point of Co_2_O_3_, an additional step was added to the procedure waiting point around 850 °C. One of the samples (H1) was synthesized with a predrying step to identify if the presence of water molecules affects the SPS. Predrying was done at 85 °C for 15 min.

**Table 1:** Sample Preparation Conditions and Final Dimensions

sample	temperature during SPS	predrying	total process time, min	heating, K/min	diameter, mm	width, mm
H1	1200	15 min at 85 °C	26.0	50–82 °C	13.7	2.02
H2	1200		26.0	50–82 °C	13.7	1.70
H3	1250		31.5	25–82 °C	13.3	1.55
H4	1300		26.5	50–82 °C	13.6	1.34

### Characterization

The phase compositions of the samples were studied by X-ray diffraction (XRD). XRD patterns were collected from bulk samples using a PANalytical Empyrean X-ray diffractometer equipped with a Cu LFF HR X-ray tube (λ = 1.5419 Å). The analysis was conducted at room temperature for 45 min. XRD patterns were postprocessed with normalization of the background. For Rietveld analysis, HighScore Plus software was used.

The morphology/composition was investigated via scanning electron microscopy (SEM) and energy-dispersive spectroscopy (EDS) analysis. The sintered sample’s microstructural and elemental compositions were studied with dispersive X-ray spectroscopy on an FEI Magellan 400 extreme high-resolution X-Max 80 silicon drift detector (Oxford Instruments).

The density of the materials was obtained by combining the measured weight of the samples and their volume, calculated from the measured dimensions of the cylindrical samples. The equation ρ = *m*/*V* was applied, where ρ is the density of the sample, g/cm^3^, *m* is the (measured) mass of the material, g, and *V* is the volume of the sample, cm^3^. The volume of the samples was estimated via the equation *V* = π·*h*·*r*^2^ (applicable for the cylinder shape), where *h* is the height of the sample, cm and *r* is the radius of the sample, cm.

Thermogravimetric analysis (TGA) and differential scanning calorimetry (DSC) were measured on NETZSCH STA 449 F3 Jupiter equipment. Measurements were done with a heating rate of 10 K/min and in air.

The transport properties, i.e., electrical conductivity and the Seebeck coefficient, were measured by the 4-probe measurement method in the temperature range of 25–500 °C using a NETZSCH SBA 458 Nemesis. During the measurements, two microheaters generated the temperature gradient in both sample directions (one sample side was heated and cooled down, and after that, the same was applied to another sample side). The scheme of the TE measurements is reported in Figure S1.

For the obtained materials, electrical conductivity and Seebeck coefficient properties were measured during heating and cooling (AC and DC, but due to the very similar results, only plots for DC data are presented). The accuracy of the properties is around 3–5%. The values for the electrical conductivity are lower than 1 S/cm, so they are around the accuracy of the measurements.

The same properties of the material during AC and DC measurements indicate that synthesized alloys are stable till 500 °C. The temperature of 500 °C was chosen to prevent the evaporation of the secondary phases.

## Results and Discussion

**Figure 1:**
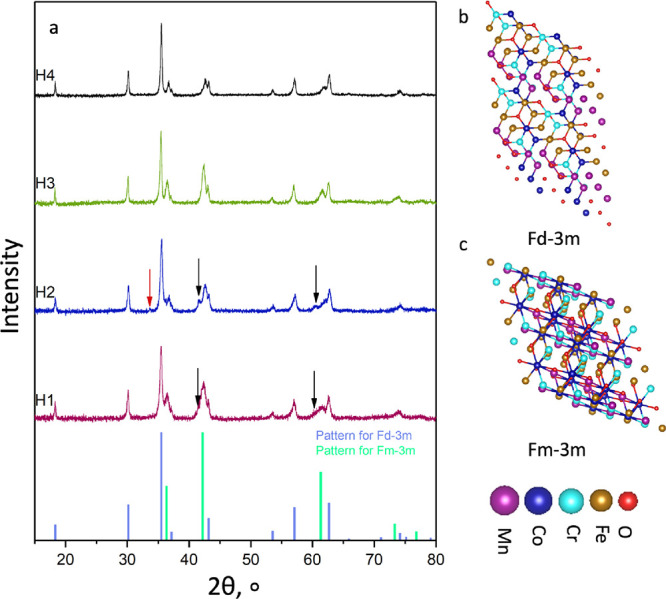
(a) X-ray diffraction patterns for the samples H1–H4. Patterns in the bottom panel for the crystal structures *Fm*3̅*m* and *Fd*3̅*m* are taken from the HighScore database under numbers ICDD 01-08306168 and ICDD 04-001-9399, respectively. Crystal structures for (b) *Fd*3̅*m* and (c) *Fm*3̅*m*. The HEA structures were created via the application of the Python algorithm with the utilization of the special quasi-random structure approach. The base unit cell data were acquired from the open database.^27^

The XRD patterns for the samples are shown in Figure 1a. All samples have the main phase with the spinel structure *Fd*3̅*m* (main peak at 35.54°). The results of the Rietveld analysis are presented in Table 2. Samples H1 and H2 have two different rock salt structures of *Fm*3̅*m* (shown as black arrows) with different parameters of the crystal structure (Table S2). Sample H2 has four phases (*Fd*3̅*m*, *Fm*3̅*m*, *Fm*3̅m*, and *R*3̅*c*). Phase *R*3̅*c* is shown as a red arrow on the graph (peak at 33.61°). In sample H2, these peaks present their maximum intensity. Sample H1 has a larger amount of phase *Fd*3̅*m*. We assume that this difference is due to the heat treatment before SPS for sample H1. None of these phases are detected in samples H3 and H4.

**Table 2:** Results of the Rietveld Analysis

	amount of the phase, %			
sample	*Fd*3̅*m*	*Fm*3̅*m*	*Fm*3̅m*	*R*3̅*c*
H1	61.1	21.3	17.6	
H2	50.8	22.1	26.5	0.5
H3	52.4	47.6		
H4	81.4	18.6		

Samples H3 and H4 are composed of the rock salt structure and spinel structure. Sample H4 has the largest amount of phase *Fd*3̅*m* (81.4%), which means that a higher temperature is needed to synthesize a single-phase material.

Crystal structures for *Fm*3̅*m* and *Fd*3̅*m* are presented in Figure 1b,c. Different reagents have different crystal structures (*Fd*3̅*m*, *Fm*3̅*m*, and *R*3̅*c*), but during the sintering process, they start to react with each other and transform into one crystal structure *Fd*3̅*m*, with the chemical formula AB_2_X_4_ (A and B, cations; X, oxygen). The formation of the main phase depends on different parameters such as temperature, time, and pressure during the sintering process. The biggest amount of the targeted phase *Fd*3̅*m* was in sample H4, which was prepared under the highest temperature during the sintering process. It means that temperature makes a major contribution to phase formation.

**Figure 2:**
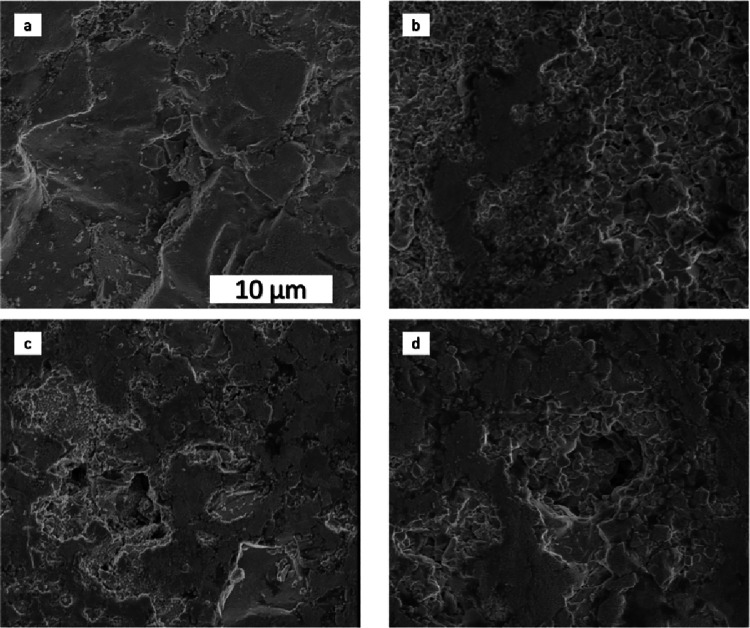
Morphology of the obtained materials with the chemical composition Co-Cr-Fe-Mn-Ni-O: (a) H1; (b) H2; (c) H3; (d) H4.

To further analyze the chemical composition and microstructure of the samples, SEM/EDS techniques were used. The morphology of the samples is presented in Figure 2. Morphology analysis indicates that the synthesized materials have an uneven and porous surface. Closer analysis shows that there are both sintered powder and self-standing agglomerates. The reason can be that some reagents (we assume Co_3_O_4_ because this oxide has the lowest melting temperature) start to melt during the SPS process and mix with other powders. During the cooling of the system, the melting stops, and reagents solidify with each other forming self-standing agglomerates. Stygar et al.^20^ showed that after 20 h of sintering in the furnace, the microstructure of the sample looked like a powder, which slightly soldered on the sides to connect with other powders. The latter is the reason for the sample to exhibit a lot of pores and a lower density of the material. The current approach leads to fewer pores on the surface. Theoretical or ideal density was calculated via the equation ρ_theory_ = (*n*·*A*)/(*V*·*N*), where *n* is the number of atoms per unit cell, *A* is atomic weight, g/mol, *V* is the volume of the unit cell, cm^3^/cell, and *N* is Avogadro’s number, 6.023 × 10^22^. The parameters for the crystal structure were taken from the HighScore database (ICDD 04-001-9399), and the atomic weight of the material was calculated based on the percentage of each element (*A* = 232 g/mol). The ideal density of the material is found to be ∼5.24 g/cm^3^. Table 3 contains information regarding the density of the obtained samples and their comparison to the ideal scenario. The obtained density of samples is lower than the theoretical. The mismatch between ideal and obtained densities may be due to the presence of defects, holes, and intrusions. This mismatch is commonly observed for the SPS-obtained materials and is considered to be negligible. It indicated that SPS is a better option to sinter powder close to the bulk material. The reason is high pressure and temperature, i.e., the sample changes its compactness due to the conditions of the obtaining process.

**Table 3:** The Density of the Obtained Materials

sample	density, g/cm^3^	ρ/ρ_theory_, %
H1	5.08	96.8
H2	5.20	99.0
H3	5.19	98.9
H4	5.10	97.1

Results of EDS elemental mapping are presented in Figure S2. In all samples, cobalt and nickel were mixed but separated from chromium, which was better mixed with iron and manganese in all samples. Samples H1 and H2 have a lower amount of manganese and iron in the scanned part of the sample. For the samples H3 and H4, Mn and Fe are homogeneously distributed and mixed with all other elements. This distribution of elements can be caused by the number of different phases in the material, and the reason for that can be duration and temperature during the sintering process. Also, it can depend on the atomic radius of the elements; Cr has the highest atomic radius, and Ni has the lowest. Probably, more time during the sintering process is needed, so there will be better interaction between all elements. The average chemical compositions for all samples are summarized in Table 4. During the preparation, different amounts of oxides were mixed to obtain high-entropy oxide (Co_3_O_4_ ≈ 34.5%, Cr_2_O_3_ ≈ 21.8%, Fe_3_O_4_ ≈ 22.9%, MnO ≈ 10.2%, and NiO ≈ 10.7%). Samples H3 and H4 have a close chemical composition due to the higher temperatures during the synthesis, and their average composition is close to the stoichiometry, which was expected (Co ≈ 15%, Cr ≈ 8.8%, Fe ≈ 9.2%, Mn ≈ 5%, and Ni ≈ 5%).

**Table 4:** EDS Results: Average Compositions (at. %)

sample	Co	Cr	Fe	Mn	Ni	O
H1	11.6	17.7	8.7	2.5	3.4	56.1
H2	15.4	18.1	6.3	0.5	4.0	55.7
H3	14.7	10.3	10.3	5.1	4.8	54.8
H4	18.0	7.4	11.4	6.2	5.5	51.5

**Figure 3:**
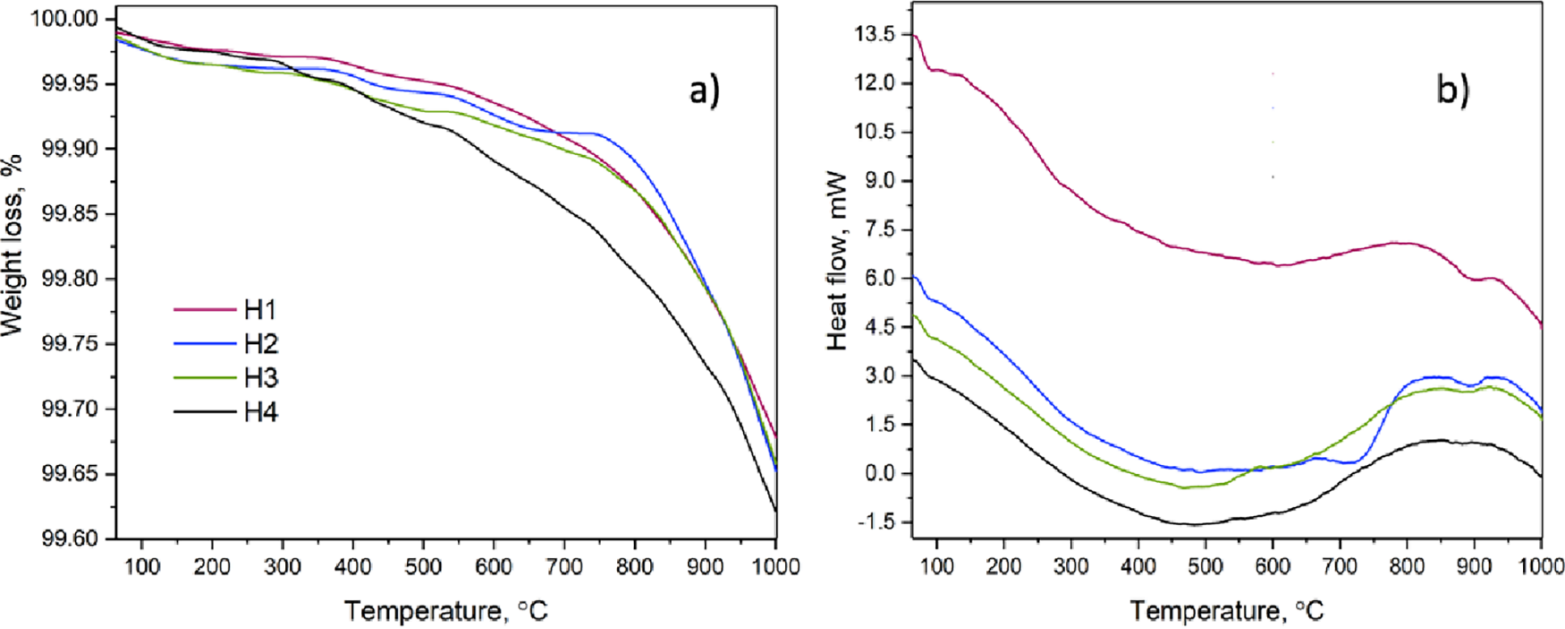
Measurements of the samples: (a) TGA and (b) DSC.

The results of the TGA and DSC measurements are presented in Figure 3. All samples have a weight loss during the whole measurement process. The final weight loss in the samples is almost the same. Sample H2 has a few additional steps in the temperature range of 400–750 °C, which is associated with the presence of phase *R*3̅*c*.

All samples have exothermal and endothermal reactions, which are clearly observable in samples H2, H3, and H4. At temperatures around 450 °C and higher, the endothermal reaction turns into exothermal. The change in the reaction can be due to the destruction of the structure, which emits energy.

**Figure 4:**
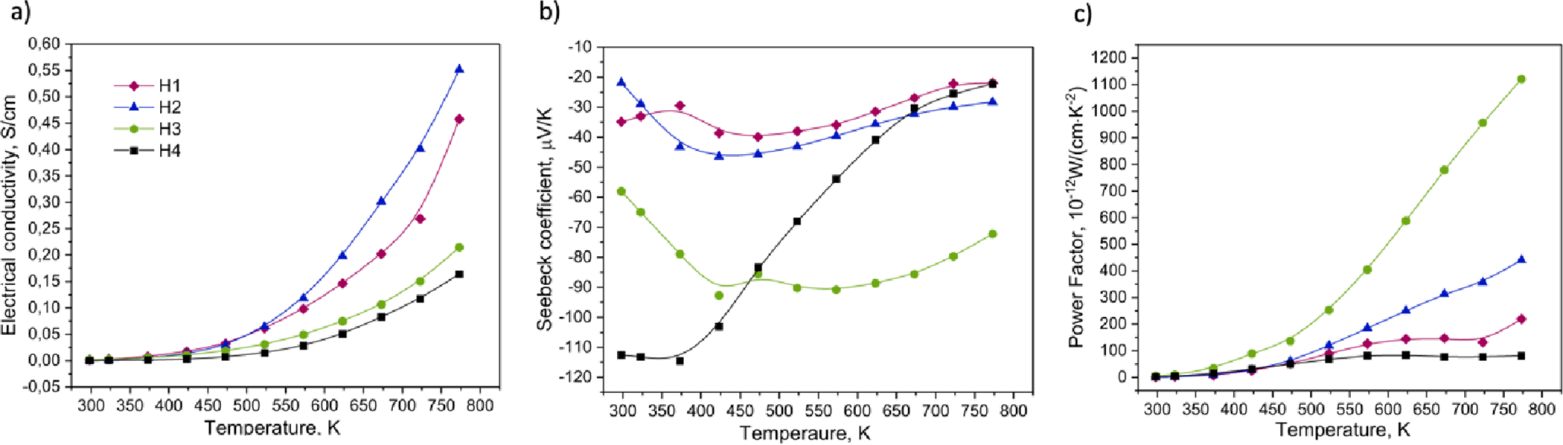
(a) Electrical conductivity, (b) Seebeck coefficient, and (c) power factor for the H1–H4 sample series.

The transport properties are reported in Figure 4. The electrical conductivity exhibits the same semiconducting behavior for all the samples, i.e., the growth of the conductivity with increasing temperatures. The increase in conductivity is almost negligible around 300 K and increases monotonically with the temperature. The sample with the highest electrical conductivity at high temperature (773 K) is sample H2 (Table 5) followed by sample H1, both annealed at 1200 °C. In these samples, the *Fm*3̅m* phase may work as the main path for the flow of charge carriers. The other two samples exhibit lower electrical conductivity values, which is probably related to the lack of the *Fm*3̅m* phase, as highlighted by XRD analysis. Electrical conductivity depends on the charge carrier concentration and their mobility (σ = *e*·*n*·μ, where *e* is the electric charge, C, *n* is the carrier concentration, m^–3^, and μ is mobility of charge carriers, m^2^/(V·s)). We assume that for all samples, the carrier concentration grows, so the mobility is falling. For samples H1 and H2, growth of the carrier concentration prevails over the decrease of the charge mobility, but for samples H3 and H4, the carrier concentration is lower than those for samples H1 and H2. The electrical properties of the materials H3 and H4 at high temperatures are close to the values of the single-phase material (σ ≈ 0.2009 S/cm), which is presented in Stygar et al.’s article,^20^ due to the high amount of the *Fd*3̅*m* phase.

**Table 5:** Electrical Conductivity and Seebeck Coefficient for the Samples at Room Temperature and 500 °C

	room temperature			773 K		
sample	σ, S/cm	*S*, μV/K	PF, 10^–12^ W/(cm·K^–2^)	σ, S/cm	*S*, μV/K	PF, 10^–12^ W/(cm·K^–2^)
H1	0.0015	–34.8552	1.7989	0.4578	–21.8931	219.4029
H2	8.2286 × 10^–4^	–21.9087	0.3949	0.5509	–28.2721	440.3744
H3	0.0018	–58.0514	5.9114	0.2148	–72.2363	1120.6624
H4	1.5839 × 10^–4^	–112.5665	2.0069	0.1641	–22.2539	81.2646

The Seebeck coefficient values (Figure 4b) for all the samples vary between −20 and −115 μV·K^–1^. The values indicate the n-type nature of the materials, i.e., electrons are the main charge carriers in all the investigated samples. Lower absolute values of the Seebeck coefficient for H1 and H2 materials are most probably due to the presence of the third phase. On the contrary, the H3 and H4 values of *S* are higher due to the purer material overall. An additional explanation could be the lower content of the unsintered powder. As observed before, with the growth of temperature, the carrier concentration also increased, which lowers the values of the Seebeck coefficient. The values of H3 are higher at elevated temperatures, whereas H4 values drop in this region. Even if obtained materials have more than one phase, the behavior of the Seebeck coefficient is close to the material with the *Fd*3̅*m* crystal structure, which was shown in the previous article.^20^

The highest PF value is for sample H3. The Seebeck coefficient is the main factor, which exhibits a quadratic contribution to PF (PF = σ · *S*^2^). Since H3 exhibits the highest values of *S* at the highest temperature range (above 475 K), this leads to the highest yield. For the single-phase material, which was presented in Stygar et al.’s article,^20^ PF was around 3395.21 × 10^–12^ W/(cm·K^–2^).

The presence of the second phase (*Fm*3̅*m*) may also have an effect on the transport properties. Its investigation would be useful to reach a single-phase system through the adjustment of SPS parameters and obtain a comparison of the TE properties between multiphase and single-phase systems. Further doping or alteration of the composition is planned to increase the yield and achieve the p-type behavior of the semiconductor material.

## Conclusions

The main goal of this work was to reduce the duration of the HEO (Co-Cr-Fe-Mn-Ni-O) synthesis process via using SPS as a much faster and more scalable alternative technique, compared to the one reported in the literature. All the obtained samples mainly exhibit the presence of the two crystal phases *Fm*3̅*m* and *Fd*3̅*m*, which is probably due to the sintering temperature. The highest amount of the spinel structure *Fd*3̅*m* is equal to 81.4%.

Low electrical conductivity values are due to only partial sintering of the precursor powder, generating powder agglomerates, which should be avoided by prolonging the sintering duration. The behavior of the Seebeck coefficient with temperature growth is close to that of the goal structure. The highest value of the Seebeck coefficient is around −115 μV·K^–1^ at room temperature, which could be explained by a high content of the *Fd*3̅*m* structure. With further growth of temperature, the value drops to 22 μV·K^–1^.

These results indicate that SPS is a fast and scalable alternative method in contrast to the conventional one. In future work, further adjustment of the SPS process to reach a single phase is planned, which may further increase the TE properties. Further steps to improve the TE functionality rely on doping and, more in general, the controlled composition of the HEA.

## Supporting Information Available

Melting temperature of precursors, parameters of the crystal structures, scheme of the electrical measurements, and EDS mapping (PDF)

## References

[ref1] Dong K.; Hochman G.; Zhang Y.; Sun R.; Li H.; Liao H. CO2 Emissions, Economic and Population Growth, and Renewable Energy: Empirical Evidence across Regions. Energy Econ 2018, 75, 180–192. 10.1016/j.eneco.2018.08.017.

[ref2] Pourkiaei S. M.; Ahmadi M. H.; Sadeghzadeh M.; Moosavi S.; Pourfayaz F.; Chen L.; Yazdi M. A. P.; Kumar R. Thermoelectric Cooler and Thermoelectric Generator Devices: A Review of Present and Potential Applications, Modeling and Materials. Energy 2019, 115849 10.1016/j.energy.2019.07.179.

[ref3] Tan G.; Zhao L. D.; Kanatzidis M. G. Rationally Designing High-Performance Bulk Thermoelectric Materials. Chem. Rev. 2016, 12123–12149. 10.1021/acs.chemrev.6b00255. 27580481

[ref4] He J.; Tritt T. M. Advances in Thermoelectric Materials Research: Looking Back and Moving Forward. Science 2017, eaak9997 10.1126/science.aak9997. 28963228

[ref5] Jaziri N.; Boughamoura A.; Müller J.; Mezghani B.; Tounsi F.; Ismail M. A Comprehensive Review of Thermoelectric Generators: Technologies and Common Applications. Energy Rep. 2020, 264–287. 10.1016/j.egyr.2019.12.011.

[ref6] Bell L. E. Cooling, Heating, Generating Power, and Recovering Waste Heat with Thermoelectric Systems. Science 2008, 321, 1457–1461. 10.1126/science.1158899. 18787160

[ref7] Chang C.; Wu M.; He D.; Pei Y.; Wu C.-F.; Wu X.; Yu H.; Zhu F.; Wang K.; Chen Y.; Huang L.; Li J.-F.; He J.; Zhao L.-D. 3D Charge and 2D Phonon Transports Leading to High Out-of-Plane ZT in n-Type SnSe Crystals. Science 2018, 360, 778 10.1126/science.aaq1479. 29773748

[ref8] Snyder G. J.; Toberer E. S. Complex Thermoelectric Materials. Nat. Mater. 2008, 105–114. 10.1038/nmat2090. 18219332

[ref9] Sallis S.; Piper L. F. J.; Francis J.; Tate J.; Hiramatsu H.; Kamiya T.; Hosono H. Role of Lone Pair Electrons in Determining the Optoelectronic Properties of BiCuOSe. Phys. Rev. B 2012, 85, 85207 10.1103/PhysRevB.85.085207.

[ref10] Barreteau C.; Pan L.; Amzallag E.; Zhao L. D.; Bérardan D.; Dragoe N. Layered Oxychalcogenide in the Bi–Cu–O–Se System as Good Thermoelectric Materials. Semicond. Sci. Technol. 2014, 29, 064001 10.1088/0268-1242/29/6/064001.

[ref11] Barreteau C.; Bérardan D.; Amzallag E.; Zhao L.; Dragoe N. Structural and Electronic Transport Properties in Sr-Doped BiCuSeO. Chem. Mater. 2012, 24, 3168–3178. 10.1021/cm301492z.

[ref12] El-Khouly A.; Novitskii A.; Adam A. M.; Sedegov A.; Kalugina A.; Pankratova D.; Karpenkov D.; Khovaylo V. Transport and Thermoelectric Properties of Hf-Doped FeVSb Half-Heusler Alloys. J. Alloys Compd. 2020, 820, 153413 10.1016/j.jallcom.2019.153413.

[ref13] Zhang Q.; Wang H.; Zhang Q.; Liu W.; Yu B.; Wang H.; Wang D.; Ni G.; Chen G.; Ren Z. Effect of Silicon and Sodium on Thermoelectric Properties of Thallium-Doped Lead Telluride-Based Materials. Nano Lett. 2012, 12, 2324–2330. 10.1021/nl3002183. 22493974

[ref14] Goldsmid H. J. Bismuth Telluride and Its Alloys as Materials for Thermoelectric Generation. Materials 2014, 2577–2592. 10.3390/ma7042577. 28788584 PMC5453363

[ref15] Yeh J. W.; Chen S. K.; Lin S. J.; Gan J. Y.; Chin T. S.; Shun T. T.; Tsau C. H.; Chang S. Y. Nanostructured High-Entropy Alloys with Multiple Principal Elements: Novel Alloy Design Concepts and Outcomes. Adv. Eng. Mater. 2004, 6, 299–303. 10.1002/adem.200300567.

[ref16] Rost C. M.; Sachet E.; Borman T.; Moballegh A.; Dickey E. C.; Hou D.; Jones J. L.; Curtarolo S.; Maria J.-P. Entropy-Stabilized Oxides. Nat. Commun. 2015, 6, 8485 10.1038/ncomms9485. 26415623 PMC4598836

[ref17] Zhang P.; Lou Z.; Qin M.; Xu J.; Zhu J.; Shi Z.; Chen Q.; Reece M. J.; Yan H.; Gao F. High-Entropy (Ca0.2Sr0.2Ba0.2La0.2Pb0.2)TiO3 Perovskite Ceramics with A-Site Short-Range Disorder for Thermoelectric Applications. J. Mater. Sci. Technol. 2022, 97, 182–189. 10.1016/j.jmst.2021.05.016.

[ref18] Banerjee R.; Chatterjee S.; Ranjan M.; Bhattacharya T.; Mukherjee S.; Jana S. S.; Dwivedi A.; Maiti T. High-Entropy Perovskites: An Emergent Class of Oxide Thermoelectrics with Ultralow Thermal Conductivity. ACS Sustainable Chem. Eng. 2020, 8, 17022–17032. 10.1021/acssuschemeng.0c03849.

[ref19] Dąbrowa J.; Stygar M.; Mikuła A.; Knapik A.; Mroczka K.; Tejchman W.; Danielewski M.; Martin M. Synthesis and Microstructure of the (Co,Cr,Fe,Mn,Ni)3O4 High Entropy Oxide Characterized by Spinel Structure. Mater. Lett. 2018, 216, 32–36. 10.1016/j.matlet.2017.12.148.

[ref20] Stygar M.; Dąbrowa J.; Moździerz M.; Zajusz M.; Skubida W.; Mroczka K.; Berent K.; Świerczek K.; Danielewski M. Formation and Properties of High Entropy Oxides in Co-Cr-Fe-Mg-Mn-Ni-O System: Novel (Cr,Fe,Mg,Mn,Ni)3O4 and (Co,Cr,Fe,Mg,Mn)3O4 High Entropy Spinels. J. Eur. Ceram. Soc. 2020, 40, 1644–1650. 10.1016/j.jeurceramsoc.2019.11.030.

[ref21] Gild J.; Zhang Y.; Harrington T.; Jiang S.; Hu T.; Quinn M. C.; Mellor W. M.; Zhou N.; Vecchio K.; Luo J. High-Entropy Metal Diborides: A New Class of High-Entropy Materials and a New Type of Ultrahigh Temperature Ceramics. Sci. Rep. 2016, 6, 1 10.1038/srep37946. 27897255 PMC5126569

[ref22] Castle E.; Csanádi T.; Grasso S.; Dusza J.; Reece M. Processing and Properties of High-Entropy Ultra-High Temperature Carbides. Sci. Rep. 2018, 8, 8609 10.1038/s41598-018-26827-1. 29872126 PMC5988827

[ref23] Zhou J.; Zhang J.; Zhang F.; Niu B.; Lei L.; Wang W. High-Entropy Carbide: A Novel Class of Multicomponent Ceramics. Ceram. Int. 2018, 44, 22014–22018. 10.1016/j.ceramint.2018.08.100.

[ref24] Krawczyk P. A.; Jurczyszyn M.; Pawlak J.; Salamon W.; Baran P.; Kmita A.; Gondek Ł.; Sikora M.; Kapusta C.; Strączek T.; Wyrwa J.; Żywczak A. High-Entropy Perovskites as Multifunctional Metal Oxide Semiconductors: Synthesis and Characterization of (Gd0.2Nd0.2La0.2Sm0.2Y0.2)CoO3. ACS Appl. Electron. Mater. 2020, 2, 3211–3220. 10.1021/acsaelm.0c00559. 33196046 PMC7660934

[ref25] Zheng Y.; Zou M.; Zhang W.; Yi D.; Lan J.; Nan C. W.; Lin Y. H. Electrical and Thermal Transport Behaviours of High-Entropy Perovskite Thermoelectric Oxides. Journal of Advanced Ceramics 2021, 10, 377–384. 10.1007/s40145-021-0462-5.

[ref26] Jiang B.; Yu Y.; Cui J.; Liu X.; Xie L.; Liao J.; Zhang Q.; Huang Y.; Ning S.; Jia B.; Zhu B.; Bai S.; Chen L.; Pennycook S. J.; He J. High-Entropy-Stabilized Chalcogenides with High Thermoelectric Performance. Science , 371 (), 830, 10.1126/science.abe129. 33602853

[ref27] Jain A.; Ong S. P.; Hautier G.; Chen W.; Richards W. D.; Dacek S.; Cholia S.; Gunter D.; Skinner D.; Ceder G.; Persson K. A. Commentary: The Materials Project: A Materials Genome Approach to Accelerating Materials Innovation. APL Mater. 2013, 011002 10.1063/1.4812323.

