# Supplementary material for: Co-Cr-Fe-Mn-Ni Oxide as a Highly Efficient Thermoelectric High-Entropy Alloy
Source: ACS Omega. 2023 Apr 14;8(16):14484–9. doi: 10.1021/acsomega.2c08278 (PMC10134248; doi:10.1021/acsomega.2c08278)
Supplement: Supplementary file 1 — ao2c08278_si_001.pdf [file ao2c08278_si_001.pdf]

Supporting information

Co-Cr-Fe-Mn-Ni oxide as a highly efficient thermoelectric high-entropy alloy

Daria Pankratova[1], Silvia Maria Giacomelli[2], Khabib Yusupov[3], Farid Akhtar[1], Alberto Vomiero[1, 4*]

[1] Department of Engineering Sciences and Mathematics, Luleå University of Technology, 97187 Luleå, Sweden

[2] Department of Industrial Engineer, Università degli Studi di Padova, Via Giovanni Gradenigo, 6a, 35131 Padova PD, Italy

[3] Department of Physics, Chemistry, and Biology, Linköping University, 581 83, Linköping, Sweden

[4] Department of Molecular Sciences and Nanosystems, Ca' Foscari University of Venice, Via Torino 155, 30172 Venezia Mestre, Italy

Table S1 – Melting temperatures for the precursors

Precursor	Melting temperature, °C
Co_3O_4	895
Cr_2O_3	2435
Fe_2O_3	1565
MnO	1945
NiO	1955
CoO	1935
Co_2O_3	895

Table S2 – Parameters of the crystal structures

Phase	a, Å	b, Å	c, Å	α, °	β, °	γ, °
Fd-3m	8.375	8.375	8.375	90	90	90
Fm-3m	4.24	4.24	4.24	90	90	90
Fm-3m*	4.312	4.312	4.312	90	90	90
R-3c	4.958	4.958	4.958	90	90	120

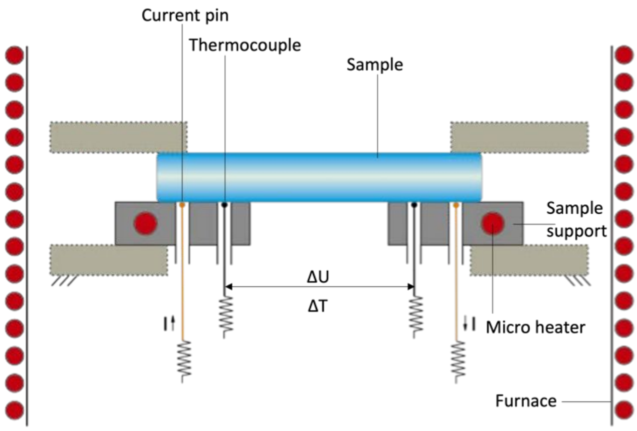

Figure S1 – Measurement setup for the SBA 458 *Nemesis*[1]

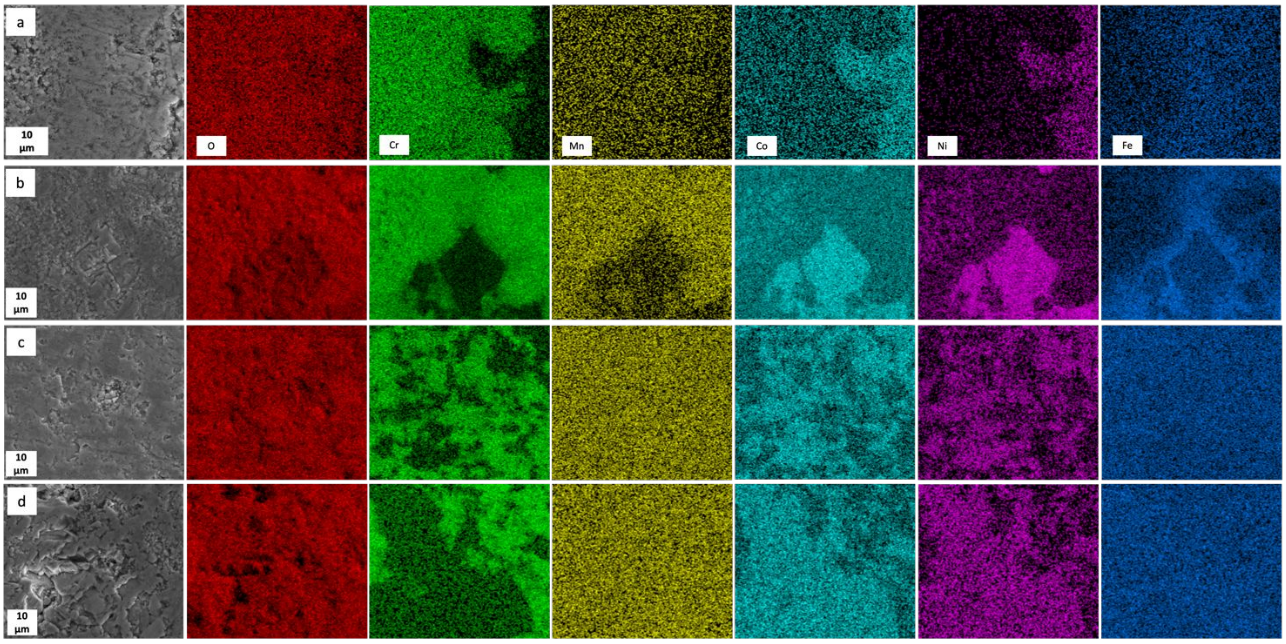

Figure S2 –EDS mapping for the Co-Cr-Fe-Mn-Ni-O samples: a) H1, b) H2, c) H3, d) H4.

References

(1) Simultaneous Determination of the Seebeck Coefficient and Electrical Conductivity-SBA 458 Nemesis Method and Technique for the Characterization of Thermoelectric Materials.
